# Quantitative Assessment of Cervical Vertebral Maturation Using Cone Beam Computed Tomography in Korean Girls

**DOI:** 10.1155/2015/405912

**Published:** 2015-03-23

**Authors:** Bo-Ram Byun, Yong-Il Kim, Tetsutaro Yamaguchi, Koutaro Maki, Woo-Sung Son

**Affiliations:** ^1^Department of Orthodontics, Dental Research Institute, Pusan National University Dental Hospital, Geumoro 20, Yangsan 626787, Republic of Korea; ^2^Department of Orthodontics, School of Dentistry, Pusan National University, Yangsan 626870, Republic of Korea; ^3^Department of Orthodontics, Biomedical Research Institute, Pusan National University Hospital, Gudeokro 179, Busan 602739, Republic of Korea; ^4^Department of Orthodontics, School of Dentistry, Showa University, Tokyo 1428555, Japan

## Abstract

This study was aimed to examine the correlation between skeletal maturation status and parameters from the odontoid process/body of the second vertebra and the bodies of third and fourth cervical vertebrae and simultaneously build multiple regression models to be able to estimate skeletal maturation status in Korean girls. Hand-wrist radiographs and cone beam computed tomography (CBCT) images were obtained from 74 Korean girls (6–18 years of age). CBCT-generated cervical vertebral maturation (CVM) was used to demarcate the odontoid process and the body of the second cervical vertebra, based on the dentocentral synchondrosis. Correlation coefficient analysis and multiple linear regression analysis were used for each parameter of the cervical vertebrae (*P* < 0.05). Forty-seven of 64 parameters from CBCT-generated CVM (independent variables) exhibited statistically significant correlations (*P* < 0.05). The multiple regression model with the greatest *R* ^2^ had six parameters (PH2/W2, UW2/W2, (OH+AH2)/LW2, UW3/LW3, D3, and H4/W4) as independent variables with a variance inflation factor (VIF) of <2. CBCT-generated CVM was able to include parameters from the second cervical vertebral body and odontoid process, respectively, for the multiple regression models. This suggests that quantitative analysis might be used to estimate skeletal maturation status.

## 1. Introduction

Knowledge of bone age is necessary in order to determine treatment timing in orthodontic and orthognathic surgery patients. Many researchers have introduced a variety of biological indicators [1–4] including chronologic age, dental development, sexual maturation, voice change, and body height [5–9]. Unfortunately, chronologic age alone is not a reliable indicator for the evaluation of skeletal maturation.

Among biological skeletal maturation indices, skeletal maturation indicators that use hand-wrist radiographs are popular and reliable approaches in orthodontia clinics. However, this method requires a hand-wrist radiographic film. There has been increased interest in the use of cervical vertebral maturation (CVM) as a replacement for the hand-wrist assessment [10–12]. Franchi et al. [11] used lateral cephalometric analysis to confirm the validity of six CVM stages as biological indicators of both somatic and mandibular skeletal maturity. Baccetti et al. [12] introduced an improved version of the CVM method for the assessment of mandibular growth. The CVM method has proven to be effective in assessing adolescent growth peaks regarding both body height and mandibular size [10–12]. However, Gabriel et al. [13] have raised issues regarding the poor inter- and intraobserver reliability of CVM, which are below 50% and 62%, respectively. They insisted that there is inherent bias in staging and that it would be necessary to use fewer vertebral bodies, employ more sensitive parameters, and avoid estimating stage based on a comparative assessment of changes between stages, in order to make the CVM analysis clearer, easier, and more applicable to the majority of patients. To achieve these conditions, Chen et al. [14] suggested a quantitative CVM (QCVM) method that used parameters from the second, third, and fourth cervical vertebrae.

Among the cervical vertebrae subjected to CVM analysis, the first (atlas) and second (axis) cervical vertebrae are atypically shaped. In particular, the second cervical vertebra contains the odontoid process, which projects upward from the body. To test the more quantitative aspects of the CVM method, Chatzigianni and Halazonetis [15] applied statistical shape analysis for the evaluation of cervical vertebral shape. In their study, morphological variations of the axis retained the same ratio of height to width as that of other vertebrae. Çokluk et al. [16] used magnetic resonance imaging (MRI) to examine the region including the occiput and the first, second, and third cervical vertebrae. They reported that the average ratio of the odontoid process to the body of the second cervical vertebra was 2 in pediatric cases and 1.8 in adult cases. In the MRI study, the remnant of dentocentral synchondrosis was used as the border between the second cervical vertebral body and its odontoid process. Çokluk et al. [16] defined the borders of the body and odontoid process of the second cervical vertebra. However, a few studies still consider the second cervical vertebral body and odontoid process to be parameters for the assessment of skeletal maturation because a lateral cephalogram is unable to demarcate the second cervical vertebral body and odontoid process separately.

Cone beam computed tomography (CBCT) could be a useful tool for the three-dimensional approach to the second cervical vertebral structure. Like MRI, CBCT is able to show the remnant of dentocentral synchondrosis in the second cervical vertebra. The images obtained from CBCT could provide another approach to the CVM method by defining the second vertebral body and odontoid process, followed by the remnant of dentocentral synchondrosis. Joshi et al. [17] have already used CBCT to assess skeletal maturation and prove the reliability of CBCT when employed with CVM. In addition, CBCT is available at a lower cost and yields high-quality data at relatively low radiation dosages; therefore, it has become used more commonly in dental practice [18].

This study aims to examine the correlation between skeletal maturation stage and parameters from CBCT-generated CVM (the odontoid process and the body of the second cervical vertebra and the bodies of the third and fourth cervical vertebrae), while simultaneously building multiple regression models that are able to estimate skeletal maturation status in Korean girls.

## 2. Material and Methods

### 2.1. Study Population

The study population included 76 patients (76 girls between 6 and 18 years of age) enrolled from July 2007 to December 2014. We examined the upper cervical spine of each patient using CBCT imaging (Pax-Zenith3D, Vatech, Seoul, Korea). Hand-wrist radiographs (PM2002CC, Planmeca, Helsinki, Finland) were used to determine skeletal maturation [19, 20]. Individuals with cleft lip and/or palate, trauma, or syndromes were excluded from the study (Table 1). The Institutional Review Board of Pusan National University Dental Hospital reviewed and approved this study (PNUDH-20104-011).

### 2.2. Skeletal Assessments

As a measure of participants' skeletal status, two investigators assessed the hand-wrist radiographs. The assessments were based on the Sempé maturation level (SML) and Fishman's skeletal maturation index (SMI). CBCT scans (field of view (FOV), 20 × 19 cm; tube voltage, 90 kVp; tube current, 4.0 mA; and scan time, 24 s) were obtained in an upright position with maximum intercuspation. Each patient's Frankfurt-horizontal (FH) plane was set parallel to the floor. The CBCT data were reconstructed using 3D imaging software (OnDemand3D, Cybermed Co., Seoul, Korea). To obtain images of the cervical vertebrae, 3D imaging software was used under the same condition (window width at 4000, window level at 1000).

### 2.3. Data Acquisition

**Figure 1:**
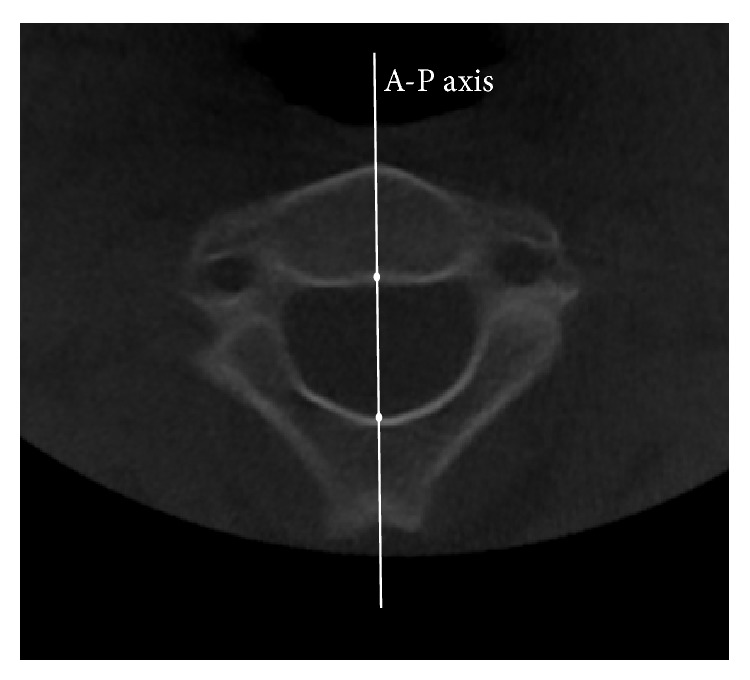
Anteroposterior (A-P) axis.

**Figure 2:**
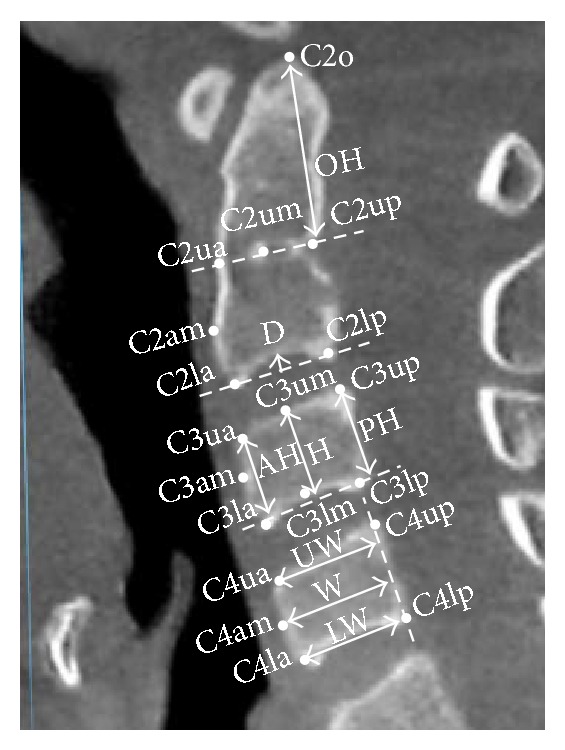
Parameters (distances) used in the analysis: OH, vertical distance of C2o to the line of C2ua and C2up; AH2–4, vertical distance of C2–4ua to the line of C2–4lp and C2–4la; H2–4, vertical distance of C2–4um to the line of C2–4lp and C2–4la; PH2–4, vertical distance of C2–4up to the line of C2–4lp and C2–4la; UW2–4, vertical distance of C2–4ua to the line of C2–4up and C2–4lp; W2–4, vertical distance of C2–4am to the line of C2–4up and C2–4lp; LW2–4, vertical distance of C2–4la to the line of C2–4up and C2–4lp; D2–4, vertical distance of C2lm to the line of C2la and C2lp.

To acquire each image, first, the deepest posterior point of the second cervical vertebral foramen and the midpoint of the second cervical vertebral body were used to generate the anterior-posterior axis in the axial view (Figure 1). After the anterior-posterior axis had been set, we adjusted the vertical axis to pass through the midpoint of the odontoid process in the coronal view. The lateral image of the cervical vertebra was obtained from the plane formed by the vertical axis and the anterior-posterior axis (Figure 2).

In this study, we adopted and modified landmarks and measurements from those described in the QCVM study by Chen et al. [14]; these parameters are defined in Figure 1 and Table 2. The border of the body of the second cervical vertebra (C2) was defined by the remnant of the dentocentral synchondrosis as visualized in the lateral image of the second cervical vertebra. For every study participant, each parameter was measured and calculated using 3D imaging software.

### 2.4. Statistical Analysis

The data were analyzed using statistical software (SPSS version 21.0 for Windows, Chicago, Il, USA). The statistical analyses included correlation coefficient analysis and multiple regression analysis with stepwise elimination. Multiple linear regression analysis was used to determine the SML for Korean girls as the dependent variable and parameters from CBCT-generated CVM as the independent variables. The intraexaminer and interexaminer reliability of the linear measurements were each checked by remeasurement of 20 randomly selected lateral images 2 weeks later; the intraclass correlation coefficients were very high (means of 0.993 and 0.990, resp.). The intraobserver and interobserver errors for the SML and SMI were evaluated using Cohen's kappa index. The intraobserver and interobserver reliability for Cohen's kappa index each demonstrated substantial agreement (means of 0.805 and 0.779, resp.).

## 3. Results

To prove that the SMI and SML are comparable, we used Pearson's correlation coefficient analysis to determine the correlation between the SMI and SML, which we then defined as the skeletal maturation status index. This value exhibited a very high coefficient 0.950 when the SML was substituted for the SMI. As a parametric method to present the degree of correlation between two variables, we used the Shapiro-Wilk test (*P* < 0.05) to confirm the normality test in the sample distribution.

The descriptive statistics of each parameter (obtained from CBCT-generated CVM) are reported in Tables 3 and 4. We calculated the correlation coefficient from these data in order to find the correlation between the SML and the parameters from CBCT-generated CVM (Table 5). Out of a total of 64 parameters, 47 exhibited statistically significant correlations (*P* < 0.05). Regression analysis with the SML (a dependent variable) and 64 parameters from CBCT-generated CVM (independent variables) were employed to build eight multiple regression models (Table 6). Among these models, the eighth multiple regression model (Table 6) featured the highest *R* ^2^ and adjusted *R* ^2^. The model with PH2/W2, UW2/W2, (OH + AH2)/LW2, UW3/LW3, D3, and H4/W4, as independent variables, exhibited the highest coefficient of determination, 0.786, indicating that approximately 78.6% of the variation in the SML could be explained by these independent variables. Aside from the significant enhancement of *R* ^2^, there was no significant multicollinearity between variables derived from regression models for the SML. We performed multicollinearity tests based on the variance inflation factor (VIF) <2, which indicated the absence of multicollinearity problems. The parameters AH3/W3 and H4/W4 had high VIF values (>2.0) in the fourth, fifth, and sixth regression models. The models with these VIFs could have significant multicollinearity problems [21] (Table 6).

## 4. Discussion

Several methods for assessing skeletal maturation status have been introduced [1–4]. The CVM method has proven to be especially useful. Mito et al. [22] and Chen et al. [23] introduced multiple regression models in order to employ CVM to determine skeletal maturation stage. Mito et al. [22] created a regression formula to obtain cervical vertebral bone age; they reported that the formula was determined from the ratios of measurements in the third cervical vertebral (C3) and fourth cervical (C4) vertebral bodies. Chen et al. [23] also developed formulas that employed regression analysis to predict mandibular length increment using cervical vertebrae and used the ratios of C3 and C4 vertebral bodies and the lower concavity angle of C2 as independent variables. However, in this study, we defined the measurements of the C2 vertebral body and the odontoid process. The dentocentral synchondrosis was considered the border between the body and the odontoid process of C2. The results showed that the ratios of the C2 body and the odontoid process were correlated very highly with skeletal maturation status (as did the bodies of the third and fourth cervical vertebrae, which were used in the previous studies) (Tables 5 and 6).

During embryonic development, the second cervical vertebra arises from five primary bone growth centers (one occurs in the body, two occur in bilateral neural arches, and the other two occur in the odontoid process) separated by synchondrotic articulations [24, 25]. The two in the odontoid process are fused at birth. The border between the odontoid process and the body of the axis vertebra is well demarcated by the dentocentral synchondrosis [26]. The bilateral neurocentral synchondroses between the bilateral neural arch and the dentocentral synchondrosis fuse at 3 to 6 years of age. After 6 years of age, the odontoid process fuses with the vertebral body and the neural arches [26]. The remnant of the dentocentral synchondrosis, which is below the superior articulating facets, appears on CBCT and MRI images as a ring. Çokluk et al. [16] compared the odontoid process-to-vertebral body ratios between children and adults, based on MRIs of the dentocentral synchondrosis. Therefore, we were able to use CBCT images to define the vertebral body and its odontoid process and expected that the odontoid process and vertebral body might be important independent variables for the regression model derived from CBCT-generated CVM.

Several studies used the third and fourth vertebral bodies and the concavity of their lower borders to assess skeletal maturation [10, 22, 23]. They reported that many ratios of the width and height of the C3 and C4 were correlated with skeletal maturation status. In the present study, 47 of 64 parameters exhibited statistically significant correlations (*P* < 0.05), similar to the findings of other previous studies (Table 5). The C2 vertebral body and odontoid process were significant independent variables in all multiple regression models. In the multiple regression model with the highest *R* ^2^, adjusted *R* ^2^, and variance inflation factors (VIF) <2, which indicated the absence of multicollinearity problems, PH2/W2, UW2/W2, (OH + AH2)/LW2, UW3/LW3, D3, and H4/W4 were independent variables. This model also exhibited the highest coefficient of determination, 0.786, indicating that these independent variables were able to explain approximately 78.6% of the SML variation.

While the ratios of the third and fourth cervical vertebrae had strong correlation coefficients in previous CVM studies, the present study added the ratio of the second cervical vertebra, which correlated strongly with CBCT-generated CVM as an indicator. Altan et al. [27] investigated the longitudinal growth of cervical vertebrae in girls from 8 to 17 years of age. The growth of the cervical vertebrae follows a somatic pattern, and they reach their final size at maturity [28]. C1 and C4 reached their peak size at approximately 11.5 years of age, and C3 reached its maximal size at 10.5 years of age. However, the growth curve for C2 did not have a distinct peak growth rate; its growth rate curve was more linear [29]. Therefore, in this study, the parameters of the C2 vertebral body and the odontoid process might be independent variables for the multiple linear regression model because it has a more linear growth pattern. Our findings indicated that the growth of C2 vertebral body and odontoid process might be a useful indicator for evaluating the stages of skeletal maturation. The multiple regression models put forth by Chen et al. [23] and Mito et al. [22] included parameters such as the height/width ratios of the third and fourth cervical vertebrae as independent variables. However, regression models in our study with AH3/H3 and H4/W4 ratios had multicollinearity problems with high VIF values (Table 6).

Similarly, the third and fourth cervical vertebrae arise from three primary ossification centers (one in the body and two in the posterior arches) [30, 31]. The shape of the bodies of third and fourth cervical vertebrae changes from wedge-shaped to rectangular and then becomes square. Their shapes grow vertically and then horizontally. The total length and height of the third and fourth cervical vertebrae have very similar incremental curves. Cervical vertebral growth occurred in the order of the upper to the lower cervical vertebrae. Therefore, these parameters might induce statistical multicollinearity problems. Many clinical studies regarding dental research have used multiple regression analysis to find correlations between the predictor and the outcome variables. However, collinearity in the statistic regression models is one possible problem with the use of multiple regression analysis. “Multicollinearity” is the statistical term for the situation in which more than two covariates are highly correlated [21]. It can distort the interpretation of statistical results from multiple regression models, increasing both inaccuracy and uncertainty. Multicollinearity should be considered in the regression model.

We used the SML as a measure of the study participants' skeletal maturation status in order to build the multiple regression models in this study. The SML comprises 1000 stages, numbered from 0 to 999; compared to 11 stages, it provides a more refined skeletal maturation status as a percentage. The SML (with stages 0 to 999) was pertinent to statistical analysis as a parametric dependent variable for multiple regression models. The present study has already confirmed its high correlation to Fishman's SMI for building multiple regression models (*P* < 0.05; correlation coefficient, 0.950).

Other CVM-based studies had large study populations that were heterogeneous regarding sex and ethnicity. Although our study population was comparatively small and homogeneous, this study had the advantage of introducing the modified CBCT-generated CVM with the defined body of the second cervical vertebra and the odontoid process from the dentocentral synchondrosis. Given this CBCT approach, the study could not have a large study population. Despite these difficulties, this study demonstrated a new application of the second cervical vertebral body and odontoid to the CBCT-generated CVM and resulted in the building of multiple regression models. We expect that sexual dimorphism may imply divergence between boys and girls during the course of ossification. Ethnicity may explain the relatively high correspondence of ossification level observed in the present study compared with other studies. Therefore, additional studies are needed to address questions regarding sexual dimorphism and ethnicity.

## 5. Conclusions

CBCT-generated CVM was able to include parameters from the second cervical vertebral body and the odontoid process, respectively, for multiple regression models. The derived multiple regression models demonstrated the potential of the newly defined parameters of the second cervical vertebra.

## Figures and Tables

**Table 1:** Descriptive statistics of study participants.

Sample size (*n* = 76)	Chronologic age (years)	Sempé maturation level (SML, %)
Mean age	14.6	83.1
Maximum	18.0	94.8
75% quartile	16.6	90.9
Median	15.3	87.9
25% quartile	13.6	82.7
Minimum	6	13.6

**Table 2:** Landmarks and measurements.

Parameter	Description
Landmarks	
C2o	The most superior point of the odontoid process
C2–4lm	The most superior points of the lower border of the bodies of C2–4
C2–4la, C2–4lp	The most anterior (a) and posterior (p) points on the lower border of the bodies of C2–4
C2–4ua, C2–4up	The most superior points of the anterior (a) and posterior (p) borders of the bodies of C2–4
C2–4um	The middle of the upper border of the bodies of C2–4
C2–4am	The middle of the anterior border of the bodies of C2–4
Ratios	
AH2–4/H2–4	Ratio of AH2–4/H2–4
H2–4/PH2–4	Ratio of H2–4/PH2–4
AH2–4/PH2–4	Ratio of AH2–4/PH2–4
UW2–4/W2–4	Ratio of UW2–4/W2–4
W2–4/LW2–4	Ratio of W2–4/LW2–4
UW2–4/LW2–4	Ratio of UW2–4/LW2–4
AH2–4/UW2–4	Ratio of AH2–4/UW2–4
H2–4/UW2–4	Ratio of H2–4/UW2–4
PH2–4/UW2–4	Ratio of PH2–4/UW2–4
AH2–4/W2–4	Ratio of AH2–4/W2–4
H2–4/W2–4	Ratio of H2–4/W2–4
PH2–4/W2–4	Ratio of PH2–4/W2–4
AH2–4/LW2–4	Ratio of AH2–4/LW2–4
H2–4/LW2–4	Ratio of H2–4/LW2–4
PH2–4/LW2–4	Ratio of PH2–4/LW2–4
OH/UW2	Ratio of OH/UW2
OH/AH2	Ratio of OH/AH2
OH/H2	Ratio of OH/H2
OH/PH2	Ratio of OH/PH2
OH/UW2	Ratio of OH/UW2
OH/W2	Ratio of OH/W2
OH/LW2	Ratio of OH/LW2
OH/PH2	Ratio of OH/PH2
(OH + AH2)/UW2	Ratio of (OH + AH2)/UW2
(OH + AH2)/W2	Ratio of (OH + AH2)/W2
(OH + AH2)/LW2	Ratio of (OH + AH2)/LW2
(OH + H2)/UW2	Ratio of (OH + H2)/UW2
(OH + H2)/W2	Ratio of (OH + H2)/W2
(OH + H2)/LW2	Ratio of (OH + H2)/LW2
(OH + PH2)/UW2	Ratio of (OH + PH2)/UW2
(OH + PH2)/W2	Ratio of (OH + PH2)/W2
(OH + PH2)/LW2	Ratio of (OH + PH2)/LW2

**Table 3:** Descriptive statistics for parameters in the C2, C3, and C4 vertebral bodies.

Parameter	C2 vertebra				C3 vertebra				C4 vertebra			
	Mean	SD	Min	Max	Mean	SD	Min	Max	Mean	SD	Min	Max
AH/H	1.07	0.05	0.96	1.20	0.99	0.06	0.83	1.13	0.98	0.07	0.80	1.15
HP/H	1.15	0.05	1.01	1.29	0.99	0.05	0.90	1.17	0.97	0.05	0.86	1.07
AH/PH	1.23	0.09	0.97	1.41	0.99	0.07	0.79	1.15	0.95	0.07	0.74	1.08
UW/W	0.82	0.06	0.70	0.93	0.88	0.05	0.77	0.97	0.90	0.05	0.77	1.06
W/LW	1.10	0.08	0.83	1.26	1.08	0.04	0.98	1.17	1.06	0.05	0.96	1.18
UW/LW	0.90	0.08	0.74	1.10	0.95	0.07	0.83	1.12	0.95	0.07	0.77	1.18
AH/UW	1.27	0.11	0.99	1.55	1.05	0.20	0.55	1.41	1.00	0.20	0.51	1.37
H/UW	1.18	0.11	0.94	1.45	1.05	0.17	0.61	1.36	1.01	0.16	0.59	1.36
PH/UW	1.03	0.11	0.81	1.27	1.06	0.18	0.57	1.46	1.05	0.18	0.61	1.50
AH/W	1.04	0.08	0.86	1.26	0.91	0.15	0.51	1.20	0.89	0.15	0.48	1.17
H/W	0.97	0.08	0.78	1.15	0.92	0.12	0.56	1.15	0.90	0.12	0.56	1.16
PH/W	0.85	0.09	0.67	1.10	0.93	0.13	0.53	1.15	0.93	0.14	0.57	1.24
AH/LW	1.14	0.13	0.87	1.53	0.99	0.17	0.54	1.32	0.94	0.16	0.51	1.25
H/LW	1.06	0.11	0.85	1.37	0.99	0.14	0.57	1.27	0.96	0.13	0.59	1.24
PH/LW	0.93	0.11	0.74	1.21	1.00	0.15	0.54	1.27	0.99	0.15	0.59	1.33
D	1.75	0.62	0.06	3.11	1.73	0.64	0.01	3.11	1.51	0.60	0.12	2.75

C2, second cervical; C3, third cervical; C4, fourth cervical; Min, minimum; Max, maximum; SD, standard deviation.

**Table 4:** Descriptive statistics for SML and parameters in the C2 vertebral body and odontoid process.

Parameters	Mean	SD	Min	Max
SML	82.02	16.20	18.30	96.50
OH/UW2	1.96	0.19	1.57	2.44
OH/AH2	1.55	0.15	1.14	2.04
OH/H2	1.67	0.16	1.28	2.10
OH/PH2	1.91	0.20	1.45	2.39
O/UW2	1.96	0.19	1.57	2.44
OH/W2	1.61	0.16	1.36	2.14
OH/LW2	1.76	0.15	1.36	2.14
(OH + AH2)/UW2	3.23	0.26	2.56	3.78
(OH + AH2)/W2	2.66	0.21	2.27	3.25
(OH + AH2)/LW2	2.90	0.24	2.23	3.50
(OH + H2)/UW2	3.15	0.26	2.56	3.74
(OH + H2)/W2	2.59	0.21	2.20	3.22
(OH + H2)/LW2	2.82	0.23	2.22	3.42
(OH + PH2)/UW2	3.00	0.26	2.47	3.59
(OH + PH2)/W2	2.46	0.22	2.10	3.20
(OH + PH2)/LW2	2.69	0.22	2.15	3.28

C2, second cervical; Min, minimum; Max, maximum; SD, standard deviation.

**Table 5:** Correlation coefficient between Sempé maturation level and parameters from CBCT-generated cervical vertebral maturation.

Parameter	Correlation coefficient	*P*	Parameter	Correlation coefficient	*P*	Parameter	Correlation coefficient	*P*	Parameter	Correlation coefficient	*P*
AH3/LW3	0.827	0.000	D4	0.734	0.000	W3/LW3	0.507	0.001	H2/W2	−0.277	0.059
AH3/W3	0.818	0.000	AH3/H3	0.732	0.000	UW3/W3	−0.502	0.001	PH2/LW2	0.275	0.061
D3	0.806	0.000	PH3/UW3	0.722	0.000	H2/LW2	0.498	0.002	OH/LW2	0.233	0.096
W2/LW2	0.803	0.000	H4/LW4	0.716	0.000	(OH + H2)/W2	−0.496	0.002	H2/UW2	0.217	0.112
AH3/UW3	0.801	0.000	PH3/W3	0.713	0.000	H2/PH2	0.474	0.003	H3/PH3	−0.206	0.125
AH4/W4	0.793	0.000	D2	0.709	0.000	AH3/PH3	0.471	0.003	OH/UW2	−0.193	0.141
AH4/UW4	0.784	0.000	PH4/LW4	0.705	0.000	AH2/UW2	0.47	0.003	OH/UW2	−0.193	0.141
H4/UW4	0.781	0.000	AH4/H4	0.692	0.000	(OH + AH2)/LW2	0.47	0.003	(OH + PH2)/UW2	−0.161	0.185
H3/LW3	0.777	0.000	AH2/LW2	0.652	0.000	UW4/W4	−0.467	0.003	H4/PH4	−0.138	0.222
AH4/LW4	0.776	0.000	UW2/W2	−0.63	0.000	PH2/W2	−0.436	0.006	UW3/LW3	−0.128	0.238
H4/W4	0.774	0.000	OH/AH2	−0.614	0.000	UW2/LW2	0.43	0.006	OH/PH2	−0.12	0.253
H3/W3	0.765	0.000	AH2/H2	0.604	0.000	(OH + AH2)/W2	−0.398	0.011	AH2/W2	0.066	0.357
H3/UW3	0.762	0.000	AH2/PH2	0.601	0.000	(OH + H2)/LW2	0.386	0.013	(OH + AH2)/UW2	0.066	0.358
PH4/UW4	0.759	0.000	AH4/PH4	0.601	0.000	OH/H2	−0.367	0.018	(OH + H2)/UW2	−0.055	0.381
PH4/W4	0.754	0.000	(OH + PH2)/W2	−0.55	0.000	UW4/LW4	−0.32	0.035	PH2/UW2	−0.039	0.415
PH3/LW3	0.738	0.000	OH/W2	−0.513	0.001	(OH + PH2)/LW2	0.286	0.053	W4/LW4	0.024	0.448

CBCT: cone beam computed tomography.

**Table 6:** The results of multiple regression analysis using the stepwise elimination method.

Multiple regression models	Independent variables	*B*	SE	ß	*t*	*P*	*R* ^2^	Adjusted *R* ^2^	VIF	1/VIF
1st	Intercept	5.750	8.037		0.715	0.477	0.562	0.556		
	AH3/W3	83.521	8.693	0.750	9.608	0.000			1.000	1.000

2nd	Intercept	53.375	13.591		3.927	0.000	0.647	0.637		
	AH3/W3	81.719	7.867	0.733	10.388	0.000			1.003	0.997
	PH2/W2	−54.228	13.080	−0.293	−4.146	0.000			1.003	0.997

3rd	Intercept	102.021	19.993		5.103	0.000	0.691	0.678		
	AH3/W3	76.606	7.584	0.687	10.102	0.000			1.051	0.951
	PH2/W2	−41.478	12.961	−0.224	−3.200	0.002			1.110	0.901
	UW2/W2	−66.514	20.999	−0.227	−3.167	0.002			1.163	0.860

4th	Intercept	95.499	19.162		4.984	0.000	0.725	0.709		
	AH3/W3	41.675	14.118	0.374	2.952	0.004			4.020	0.249
	PH2/W2	−48.406	12.567	−0.261	−3.852	0.000			1.152	0.868
	UW2/W2	−66.554	19.985	−0.227	−3.330	0.001			1.163	0.860
	H4/W4	49.024	17.030	0.363	2.879	0.005			3.972	0.252

5th	Intercept	76.845	19.081		4.027	0.000	0.758	0.740		
	AH3/W3	28.671	13.984	0.257	2.050	0.044			4.426	0.226
	PH2/W2	−64.394	12.956	−0.348	−4.970	0.000			1.374	0.728
	UW2/W2	−68.148	18.872	−0.232	−3.611	0.001			1.163	0.860
	H4/W4	51.692	16.099	0.382	3.211	0.002			3.984	0.251
	(OH + AH2)/LW2	14.811	4.823	0.223	3.071	0.003			1.485	0.673

6th	Intercept	65.500	19.341		3.387	0.001	0.774	0.753		
	AH3/W3	15.279	15.001	0.137	1.018	0.312			5.361	0.187
	PH2/W2	−61.331	12.709	−0.331	−4.826	0.000			1.392	0.718
	UW2/W2	−57.274	19.086	−0.195	−3.001	0.004			1.252	0.799
	H4/W4	53.176	15.708	0.393	3.385	0.001			3.992	0.251
	(OH + AH2)/LW2	15.836	4.726	0.239	3.351	0.001			1.501	0.666
	D3	4.444	2.079	0.176	2.138	0.036			1.999	0.500

7th	Intercept	64.128	19.300		3.323	0.001	0.770	0.753		
	PH2/W2	−63.747	12.490	−0.344	−5.104	0.000			1.344	0.744
	UW2/2	−57.027	19.090	−0.194	−2.987	0.004			1.252	0.799
	H4/W4	65.587	9.915	0.485	6.615	0.000			1.590	0.629
	(OH + AH2)/LW2	17.357	4.485	0.262	3.870	0.000			1.351	0.740
	D3	5.328	1.889	0.211	2.820	0.006			1.651	0.606

8th	Intercept	96.052	23.595		4.071	0.000	0.786	0.767		
	PH2/W2	−62.449	12.154	−0.337	−5.138	0.000			1.347	0.742
	UW2/W2	−54.843	18.581	−0.187	−2.952	0.004			1.256	0.796
	H4/W4	63.270	9.693	0.468	6.527	0.000			1.608	0.622
	(OH + AH2)/LW2	16.076	4.397	0.242	3.656	0.001			1.374	0.728
	D3	5.593	1.840	0.221	3.039	0.003			1.658	0.603
	UW3/LW3	−31.028	13.910	−0.129	−2.231	0.029			1.047	0.955

*B*, unstandardized regression coefficient; SE, standard error of *B*; ß, standardized regression coefficient; VIF, variance inflation factor.
